# Traditional Chinese medicine enema for acute chronic liver failure: A protocol of systematic review and meta-analysis of randomized clinical trials

**DOI:** 10.1097/MD.0000000000022585

**Published:** 2020-10-09

**Authors:** Yueqiao Chen, Yehao Luo, Fenglan Wu, Nansheng Liao, Qinglan Shi, Dewen Mao

**Affiliations:** a Guangxi University of Traditional Chinese Medicine; b The First Affiliated Hospital of Guangxi University of Chinese Medicine, Nanning, Guangxi Province, China.

**Keywords:** acute chronic liver failure, enema, meta-analysis and systematic review, protocol, traditional Chinese medicine

## Abstract

**Background:**

Acute chronic liver failure (ACLF) is the most common type of liver failure. The clinical symptoms are complex and changeable, the treatment is difficult and the fatality rate is high. It has become an urgent problem to actively seek effective treatment means and improve the clinical efficacy of ACLF patients. Studies have shown that decreased intestinal barrier function and bacterial endotoxin translocation in ACLF patients are considered to be the key causes of enterogenic endotoxemia, and traditional Chinese medicine enema has certain advantages in adjuvant treatment of this disease. However, due to the lack of evidence, there is no specific method or suggestion, so it is necessary to carry out systematic evaluation on Traditional Chinese medicine enema for ACLF and provide effective evidence for further research.

**Methods:**

We will search the following electronic databases from their inception to July 2020: Electronic database includes PubMed, Embase, Cochrane Library, Chinese Biomedical Database WangFang, VIP medicine information, and China National Knowledge Infrastructure. Primary outcomes: survival rates, TCM syndrome score. Secondary outcomes: liver function (alanine aminotransferase, aspartic acid amino transferase, total bilirubin), blood coagulation function (prothrombin activity), adverse events. Data will be extracted by 2 researchers independently, risk of bias of the meta-analysis will be evaluated based on the Cochrane Handbook for Systematic Reviews of Interventions. All data analysis will be conducted by data statistics software Review Manager V.5.3. and Stata V.12.0.

**Results:**

The results of this study will systematically evaluate the effectiveness and safety of Traditional Chinese medicine enema for ACLF.

**Conclusion:**

The systematic review of this study will summarize the currently published evidence of Traditional Chinese medicine enema for ACLF to further guide its promotion and application.

## 1 Introduction

Acute chronic liver failure (ACLF) is based on chronic liver disease, acute decompensation and liver failure occurred in a short period of time.^[1]^ Liver failure caused by hepatitis B virus (HBV) is the most common, in which ACLF as the main clinical manifestation, accounting for 80% to 90% in China.^[2]^ It has high fatality rate, many complications, and great difficulty in treatment.^[3,4]^ Generally, there is no particularly effective treatment for patients with ACLF, and liver transplantation is the ultimate effective treatment.^[5]^ However, the disease control effect after standardized treatment by western medicine is poor, which is difficult to meet the clinical needs.^[6]^

In recent years, Traditional Chinese medicine enema has been widely used in clinical and experimental studies of ACLF, it has been shown to have some advantages in protecting liver function and delaying disease progression.^[7]^ As a form of Chinese medicine adjuvant therapy, the traditional Chinese medicine enema has been used to improve symptoms in ACLF patients, but its effectiveness and safety have not yet reached a definitive conclusion. Therefore, this research intends to adopt the method of system evaluation and meta-analysis of the traditional Chinese medicine enema for ACLF to evaluate the efficacy and safety.

## 2 Methods

### 2.1 Study registration

The protocol of the systematic review has been registered.

Registration: OSF Preregisration 29 August, 2020. https://osf.io/8xazf. This systematic review protocol will be conducted and reported strictly according to Preferred Reporting Items for Systematic Reviews and Meta-Analyses^[8]^ statement guidelines, and the important protocol amendments will be documented in the full review.

### 2.2 Inclusion and exclusion criteria for study selection

#### 2.2.1 Inclusion criteria

Inclusion criteria are all randomized controlled trials (RCTs), which main treatment of ACLF is traditional Chinese medicine enema or Traditional Chinese medicine enema with oral Chinese medicine decoction. The language of the trials to be included only Chinese or English.

#### 2.2.2 Exclusion criteria

Following studies will be excluded

1. Repeated publications
2. Review of literature and cases
3. Animal studies
4. Incomplete literature
5. Non-RCTs

### 2.3 Types of participants

The types of subjects included patients diagnosed with HBV-ACLF, regardless of their degree and possible complications. All patients were treated with traditional Chinese medicine enema or Traditional Chinese medicine enema with oral Chinese medicine decoction. Due to the different pathogenesis and mechanism, we will exclude patients with HAV, HCV, HDV, HEV, and HIV infection, hepatocellular carcinoma, or those who had undergone surgery, and those who had previously received antiviral therapy with nucleoside (acid) analogs.

### 2.4 Interventions and controls

Interventions included treatment with traditional Chinese medicine enema or Traditional Chinese medicine enema with oral Chinese medicine decoction. The control group only received conventional western medicine treatment. The routine treatment of each RCT may not be identical, but the use of Traditional Chinese medicine enema or Traditional Chinese medicine enema with oral Chinese medicine decoction is the only difference between intervention and control.

### 2.5 Types of outcome measures

#### 2.5.1 Main outcomes

1. Survival rates;
2. TCM syndrome score

#### 2.5.2 Additional outcomes

1. alanine aminotransferase;
2. aspartic acid amino transferase;
3. total bilirubin;
4. prothrombin activity.

### 2.6 Search methods

#### 2.6.1 Search resources

**Figure 1:**
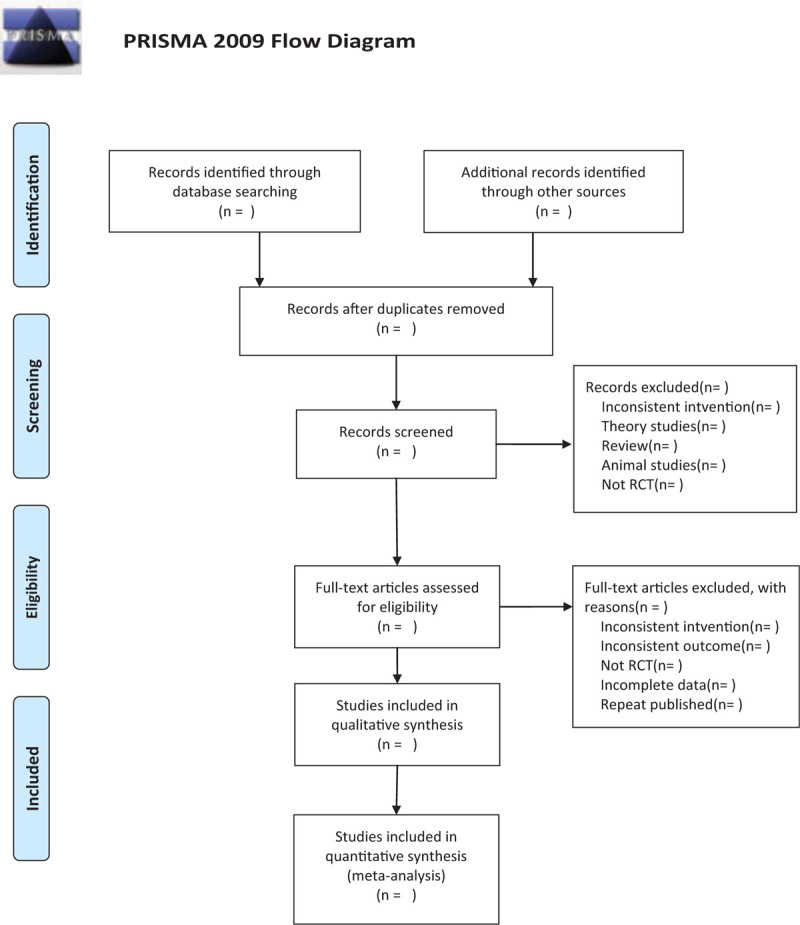
The research flowchart. This figure shows the identification, screening, eligibility, and included when we searching articles.

We will search the following electronic databases from their inception to July 2020: Electronic database includes PubMed, Embase, Cochrane Library, Chinese Biomedical Database WangFang, VIP medicine information, and China National Knowledge Infrastructure. (Fig. 1) The research flowchart.

#### 2.6.2 Search strategies

The following MeSH terms and their combinations will be searched:

(1) Traditional Chinese medicine enema or Traditional Chinese medicine enema with oral Chinese medicine decoction;
(2) RCT OR RCTs;
(3) Acute Chronic Liver Failure.

The search strategy for PubMed is shown in (Table 1). Other electronic databases will be used in the same strategy.

**Table 1:**
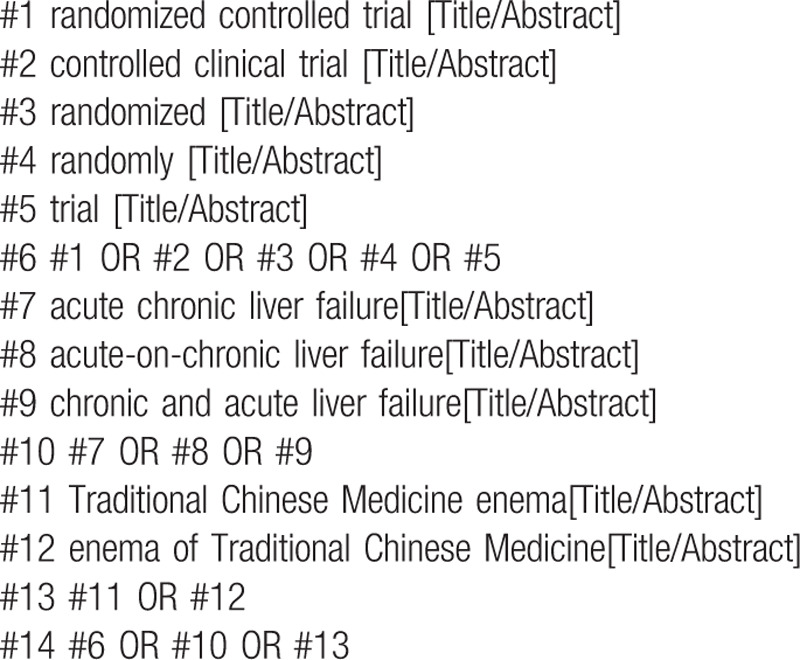
Search strategy in PubMed database.

### 2.7 Data collection and analysis

#### 2.7.1 Studies selection

There will be 2 researchers (YC and FW) carry out the selection of research literature independently using NoteExpress software. We will first make the preliminary selection by screening titles and abstracts. Second, we will download full text of the relevant studies for further selection according to the inclusion criteria. If there is any different opinion, 2 researchers will discuss and reach an agreement. If a consensus could not be reached, there will be a third researcher (QS) who make the final decision. The details of selection process will be displayed in the Preferred Reporting Items for Systematic Reviews and Meta-Analyses flow chart.

#### 2.7.2 Data extraction

Two researchers (NL and YL) will read all the included text in full, and independently extract the following information:

(1) general information, including trial name and registration information;
(2) trial characteristic, including trial design, location, setting, and inclusion/exclusion criteria;
(3) the characteristics of the participants, including age, race/ethnicity, course of illness, and so on.
(4) details of intervention, including acupoints, time of intervention, course of treatment, time of single treatment, and so on;
(5) details of comparison interventions;
(6) outcomes as described under type of outcome measure section.

If we could not reach an agreement, a third researcher (DM) would make the final decision. One researcher (YC) would contact the corresponding author by telephone or e-mail for more information when the reported data were insufficient or ambiguous.

#### 2.7.3 Assessment of risk of bias

All the included studies will be evaluated based on the guidelines of Cochrane Handbook for Systematic Reviews of Interventions.^[9]^ The quality of each trial will categorized into “low,” “unclear,” or “high” risk of bias according to the following items: adequacy of generation of the allocation sequence, allocation concealment, blinding of participants and personal, blinding of outcome assessors, incomplete outcome data, selected reporting the results and other sources of bias (such as comparable baseline characteristic, inclusion and exclusion criteria).

#### 2.7.4 Assessment of reporting biases

Reporting biases and small-study effects will be detected by funnel plot and Egger test if there are 10 more studies included in this Meta-analysis. For Egger test, *P*-value of <.10 was considered to indicate the exist of reporting biases and small study effects.

#### 2.7.5 Data analysis

We used Revman 5.3 software provided by the Cochrane collaboration to analyze the data. Binary outcomes will be summarized using risk ratio with 95% confidence interval for relative effect. Continuous outcomes will be summarized by using weighted mean difference with 95% confidence interval. We will use random-effect model for meta-analysis in this review according to research recommendations.^[10]^

Statistical heterogeneity will be assessed by *X*^2^ and *I*^2^ statistical tests. Where *P*-value ≥ .1 and *I*^2^ ≤ 50%, there is no obvious statistical heterogeneity among the studies. On the contrary, where *P*-value < .1 or *I*^2^ > 50% indicates a considerable heterogeneity. Meta-analysis will be performed when the statistical heterogeneity is acceptable (*P*-value ≥ .1 and *I*^2^ ≤ 50%), otherwise, subgroup analysis will be applied to explore the influence of potential factors on the outcome measures. We will conduct sensitivity analyses by omitting studies one by one in order to probe the impact of an individual study. If a meta-analysis cannot be performed, we will conduct descriptive analysis instead.

#### 2.7.6 Patient and public involvement

This is a meta-analysis study based on previously published data, so patient and public involvement will not be included in this study.

#### 2.7.7 Ethics and dissemination

Ethical approval will not be required as this is a protocol for systematic review and meta-analysis. The findings of this study will be disseminated to a peer-reviewed journal and presented at a relevant conference.

#### 2.7.8 Evidence assessed

The quality of evidence for this study will be assessed by “Grades of Recommendations Assessment, Development, and Evaluation (GRADE)” standard established by the World Health Organization and international organizations.^[11]^ To achieve transparency and simplification, the quality of evidence is divided into 4 levels in GRADE system: high, medium, low, and very low. We will employ GRADE profiler 3.2 for analysis.^[12]^

## 3 Discussion

ACLF has always been one of the key and difficult points in medical research, because of its high morbidity and mortality. The most common hepatitis B-related ACLF in China is caused by HBV infection.^[13]^ The patient has a long history of basic hepatitis B, and the liver cells are constantly damaged, which induces a large number of pathogenic factors accumulation and internal environment disorder. It leads to a large number of hepatocyte apoptosis. The survival number, regeneration ability, and environment of hepatocytes determine whether the patients can survive or not.^[14,15]^ The main causes of liver injury in the West are alcohol intake, infection and so on, which is mainly alcoholic liver disease.^[16]^ The current treatment focuses on protecting living hepatocytes, promoting their regeneration, and improving the cellular environment.^[17]^

However, these are not enough to reduce the mortality of ACLF. Although artificial liver and liver transplantation are now become the most effective treatment methods, the problems such as high cost, liver source scarcity and patient rejection have not been solved. Therefore, these 2 most effective methods have not been carried out in all patients.^[18,19]^ A growing body of research have shown that TCM enema can improve the clinical efficacy and quality of life of HBV-ACLF patients. Intestinal endotoxemia is the main pathogenesis of hepatocyte necrosis, so that reducing intestinal endotoxemia and avoiding intestinal infection is the key to the treatment of the disease.^[20,21]^ Traditional Chinese medicine enema can prevent the production and absorption of intestinal toxins, accelerate the metabolism of intestinal toxins, reduce the occurrence and development of intestinal endotoxemia, so as to slow down the symptoms of HE and enhance patients’ immunity and other unique functions and advantages.

## Author contributions

**Conceptualization:** Yueqiao Chen.

**Data curation:** Yueqiao Chen, Yehao Luo.

**Formal analysis:** Yehao Luo, Qinglan Shi.

**Funding acquisition:** Dewen Mao.

**Investigation:** Nansheng Liao.

**Methodology:** Nansheng Liao.

**Project administration:** Dewen Mao.

**Resources:** Fenglan Wu.

**Software:** Fenglan Wu.

**Supervision:** Dewen Mao.

**Writing – original draft:** Yueqiao Chen, Yehao Luo.

**Writing – review & editing:** Dewen Mao.

## Abbreviations

Abbreviations: ACLF = acute chronic liver failure, RCT = randomized controlled trial.
